# Sensory Entrainment Mechanisms in Auditory Perception: Neural Synchronization Cortico-Striatal Activation

**DOI:** 10.3389/fnins.2016.00361

**Published:** 2016-08-10

**Authors:** Catia M. Sameiro-Barbosa, Eveline Geiser

**Affiliations:** ^1^Service de Neuropsychologie et de Neuroréhabilitation, Centre Hospitalier Universitaire VaudoisLausanne, Switzerland; ^2^The Laboratory for Investigative Neurophysiology, Department of Radiology, Centre Hospitalier Universitaire VaudoisLausanne, Switzerland; ^3^Department of Brain and Cognitive Sciences, McGovern Institute for Brain Research, Massachusetts Institute of TechnologyCambridge, MA, USA

**Keywords:** entrainment, neural oscillations, striatum, auditory, regularity, beat, phase-locking, predictive coding

## Abstract

The auditory system displays modulations in sensitivity that can align with the temporal structure of the acoustic environment. This sensory entrainment can facilitate sensory perception and is particularly relevant for audition. Systems neuroscience is slowly uncovering the neural mechanisms underlying the behaviorally observed sensory entrainment effects in the human sensory system. The present article summarizes the prominent behavioral effects of sensory entrainment and reviews our current understanding of the neural basis of sensory entrainment, such as synchronized neural oscillations, and potentially, neural activation in the cortico-striatal system.

## Introduction

Two pendulum clocks positioned on the same table synchronize over time; this is a process called “entrainment” (Huygens, 1893). Many scientific fields have adopted this terminology for conditions in which two dynamic systems align. This review focuses on *sensory entrainment*, that is, the behaviorally observed temporal alignment of the sensory system with its environment. In everyday situations, motor actions, such as clapping in synchrony with music or alignment of walking pace in a group of people, are the result of sensory entrainment (for a review, see Ross and Balasubramaniam, 2014; see Merchant et al., 2015). However, sensory entrainment is relevant beyond motor behavior. Our sensory environment is unimaginable without its temporal structure. Tuning in to this temporal structure is thought to be a fundamental mechanism required for efficient auditory and speech perception (for a review see Giraud and Poeppel, 2012; Golumbic et al., 2013; Zoefel and VanRullen, 2015). Such sensory entrainment is, for example, evidenced through facilitated sensory perception in the context of temporal regularity (Jones et al., 2002; Geiser et al., 2012). We review neural correlates that potentially underlie the behaviorally observed alignment of the sensory system to a temporally regular or quasi regular environment.

## Behavioral evidence of sensory entrainment

The behavioral effects of sensory entrainment are typically shown in the context of temporally regular, ideally isochronous, environmental stimulation in which the occurrence of the next sensory input can be temporally predicted. For example, to measure sensory-motor synchronization, listeners tap to temporally regular auditory stimulation (Nozaradan et al., 2015). Synchronization to auditory cues is more precise than to visual cues (Hove et al., 2013), although synchronization to visual and even tactile cues is also used to measure entrainment (Lange and Roeder, 2006; Fernandez Del Olmo et al., 2007; Elliott et al., 2010, 2011; Ruspantini et al., 2011). Sensory-motor synchronization tasks include not only sensory but also motor entrainment.

Pure sensory entrainment is measured in perceptual tasks. These tasks typically show facilitated perception of stimuli when they are presented in a temporal context that allows entrainment compared to a context that does not allow entrainment. In the auditory domain, auditory temporal regularity, compared to temporal irregularity, results in faster reaction times to tones in various tasks (Lange, 2009; Rimmele et al., 2011), as well as better discrimination of differences in pitch (Jones et al., 2002), intensity (Geiser et al., 2012), and duration (Barnes and Jones, 2000; McAuley and Jones, 2003). Similar effects are observed in the visual domain (Rohenkohl et al., 2012; Marchant and Driver, 2013) and cross-modally, as in cases of auditory regular temporal grids facilitating saccadic eye movement (Bolger et al., 2013; Miller et al., 2013) and improving visual word recognition and discrimination (Bolger et al., 2013; Brochard et al., 2013) and of rhythmic movement facilitating sound perception (Morillon et al., 2014). Sensory facilitation is even observed against competing task demands (Cutanda et al., 2015). Most importantly, sensory entrainment effects are observed not only when the target stimulus is presented in the context of temporal regularity but also when temporal regularity precedes the target stimulus and the target appears at a predictable point in time as defined by the preceding sequence (Ellis and Jones, 2010; Sanabria et al., 2011; Cason and Schön, 2012; Sanabria and Correa, 2013; Cason et al., 2015). For example, sound signal detection is modulated at the rate of a previously presented amplitude modulated signal (Hickok et al., 2015). Thus, a variety of experimental tasks show the temporal context sensitivity of the sensory system, indicating facilitated perception through temporal regularity. Critically, sensory entrainment is behaviorally evidenced by the internal perpetuation of previously entrained excitability of the sensory system.

Outside of the research context, strictly regular, isochronous stimulation is the exception; it is found in music, in which temporal regularity is a defining feature (Geiser et al., 2014). However, there is emerging evidence that auditory sensory entrainment is present even in the absence of strict temporal regularity. Although behavioral effects are greatest in the context of temporal isochrony, sound perception is facilitated by varying degrees of temporal expectation (Herrmann et al., 2016). The capacity of the sensory system to detect and to synchronize to the average frequency of a stream of sounds and to perpetuate this synchronization, resulting in temporal predictions, is one of the preconditions allowing the use of entrainment for processing natural stimuli such as speech.

## Neural correlates of sensory entrainment

**Figure 1:**
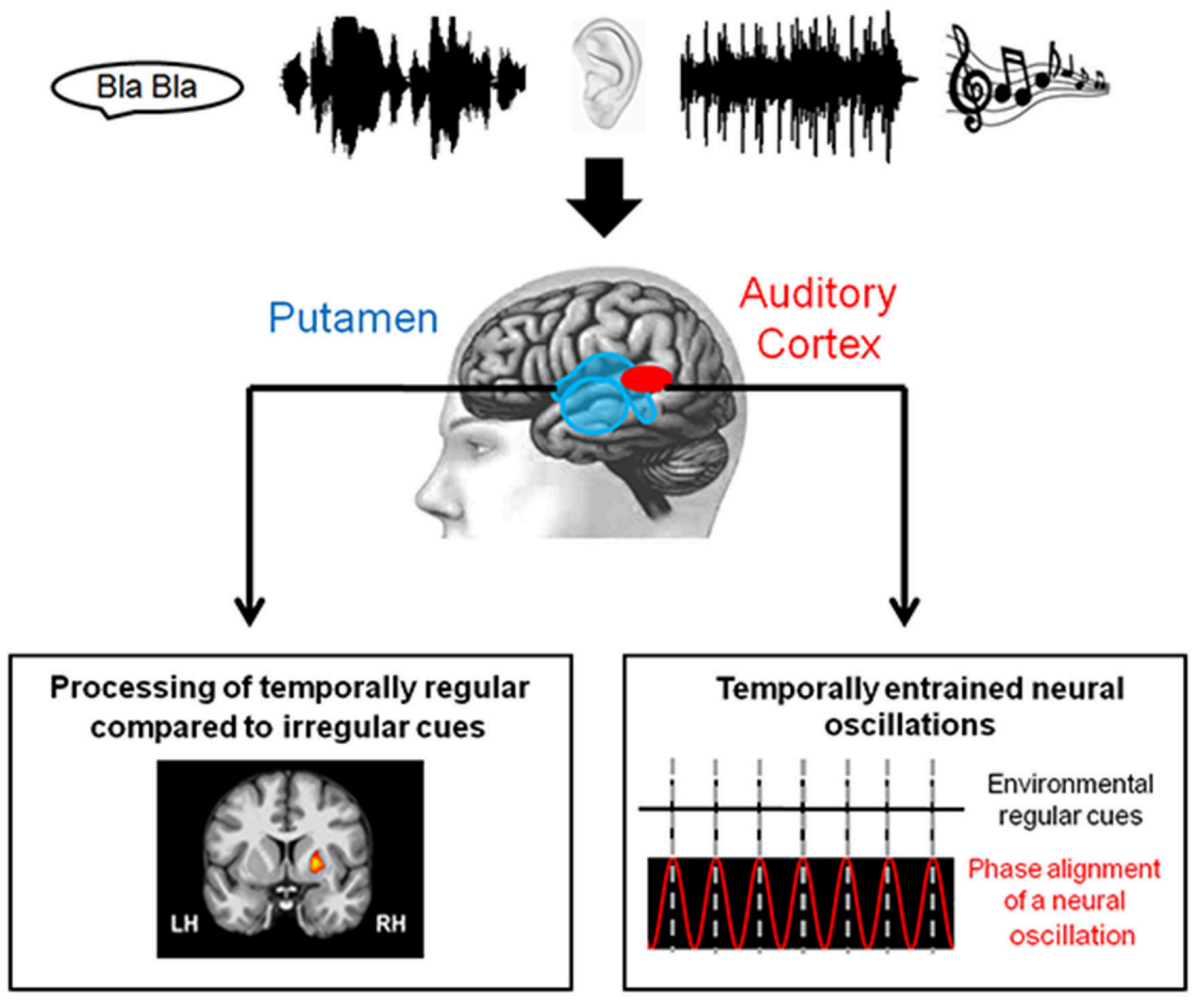
**Schematic illustration of the neural correlates of sensory entrainment**. Temporally structured auditory signals reach the sensory system (e.g., in the forms of speech and music). Neural correlates of sensory entrainment include synchronization of neural oscillations in the sensory cortices (Gross et al., 2013; Lakatos et al., 2013) and activation in the putamen (Geiser et al., 2012) (Figures adapted from Calderone et al., 2014 and Geiser et al., 2012).

The temporal context in which sounds are perceived influences neural activity. Although attention might have a modulatory effect (Hsu et al., 2014), event-related potentials (ERPs) are typically attenuated in the context of temporal regularity (Lange, 2009; Schmidt-Kassow et al., 2009; Lecaignard et al., 2015). Effects of temporal regularity are observed in the auditory N1 (Lange, 2009, 2010; Costa-Faidella et al., 2011; Rimmele et al., 2011; Sanabria and Correa, 2013) and its electromagnetic correlate N1m (Okamoto et al., 2013). Moreover, the reduction in N1 amplitude to isochronously presented tones shows the suppression of early signals, indicating a modulation of activation in secondary auditory cortices, namely the planum temporale (PT), through temporal regularity (Costa-Faidella et al., 2011). The sensitivity of sensory responses in the PT to temporal regularity is paralleled in an fMRI study on speech regularity, in which activation in the PT was modulated by temporal regularity (Geiser et al., 2008). Such modulation of neural activation by temporal regularity in primary and secondary cortices could be the result of sensory entrainment. Two mechanisms underlying sensory entrainment have been suggested, both of which may or may not be independent from each other: (1) synchronized neural oscillations in sensory and motor cortices and, potentially, (2) cortico-striatal brain activation (Figure 1). The neural correlates supporting these suggestions are reviewed in the following sections.

The first neural correlate of sensory entrainment is *synchronized neural oscillation*. Neuronal populations in the living brain show intrinsic fluctuations of excitability at the level of the cell membrane (Fiser et al., 2004; Lakatos et al., 2005). These fluctuations can be measured as periodic waves intracranially or on the scalp, via local field potentials or electroencephalograms, respectively. They can be characterized by their frequency, amplitude, and phase and are defined as delta (2–4 Hz), theta (4–8 Hz), alpha (8–12 Hz), beta (12–30 Hz), and gamma (30–100 Hz) bands. Neural oscillations typically synchronize across frequency bands, as has been shown in the auditory (Lakatos et al., 2005, 2013) and visual cortices (Lakatos et al., 2008). This hierarchical cross-frequency coupling (Schroeder and Lakatos, 2009) is suggested to influence neuronal interactions (Womelsdorf et al., 2007, for a review, see Fries, 2015). Importantly, intrinsic neural oscillations display the ability to phase-lock and thus entrain to external stimulation. This neuronal entrainment through phase-locking is observed in the visual (Montemurro et al., 2008), auditory (Luo and Poeppel, 2007; Besle et al., 2011), and somatosensory (Langdon et al., 2011; Ross et al., 2013) cortices, as well as cross-modally (Luo et al., 2010; Power et al., 2012). Thus, periodic neural oscillations synchronize to external stimulation within and across modalities.

The intrinsic oscillatory state of neuronal activity can affect whether a sensory cue is detected. Both a change in amplitude (power modulation) and the point in the cycle of a neural oscillation (phase) can influence target detection in the visual (Busch et al., 2009; Mathewson et al., 2009) and the auditory domains (Ng et al., 2012). Because the intrinsic oscillatory state can influence perception, entrained oscillations should likewise facilitate perception. Indeed, the phase of entrained neural delta oscillation predicts sound gap detection (Henry and Obleser, 2012; Henry et al., 2014). Thus, there is a strong link between the intrinsic or entrained oscillatory state of neural activity and behavioral performance.

Some components of neural oscillations, namely aspects of beta-band oscillations, seem to underlie the predictive or sustentative aspect of sensory entrainment. Synchronization of neural activity to auditory cues has been observed most strongly in the low frequencies, particularly the delta and theta frequency bands (Kayser et al., 2009; Howard and Poeppel, 2012; Ding et al., 2014), but also in higher frequencies, including the beta, and gamma frequency bands (Snyder and Large, 2005; Fujioka et al., 2012). Beta power decreases rapidly after each tone and increases before the next tone in the context of temporal regularity. Importantly, the increase depends on the tempo of the presented stimuli, with a rapid increase for fast tempi, and a slower increase for slower tempi (Fujioka et al., 2012). Moreover, when an expected stimulus is omitted, the decrease in beta power is absent, but the increase before the next tone is nevertheless present (Fujioka et al., 2009). Both findings indicate that the increase in beta power is not simply following amplitude modulations in the entraining stimulus but might represent the endogenous encoding of the predicted time interval. This modulation of the beta band by passive listening to isochronous sounds has been replicated in adults (Fujioka et al., 2015) and in children (Cirelli et al., 2014; Etchell et al., 2016). Thus, although evidence linking the predictive nature of beta-band modulations to behavior is still missing, existing electrophysiological evidence supports the idea that beta-band activity carries predictive value in the context of sensory entrainment.

Not only do neural oscillations in the sensory cortex entrain to auditory stimuli, such entrainment is also observed in other areas of the brain (i.e., motor-related brain regions). Sensorimotor cortices (the precentral and postcentral gyri), anterior cingulate cortex, cerebellum, inferior-frontal gyrus, supplementary motor area (Fujioka et al., 2012), and medial and lateral premotor cortex displayed modulation of beta oscillation in response to an external stimulation (Fujioka et al., 2015). While beta modulation in motor regions is frequently observed during movement (for review, see Khanna and Carmena, 2015), the beta activity reported here is observed in the absence of movement and must therefore relate to the temporal processing of sensory stimuli, potentially involving predictive mechanisms. It is, however, an open question whether beta oscillation in motor-related brain regions can have a predictive value, thus underlying sensory entrainment, as is assumed for the beta oscillation in the sensory cortex.

In response to more ecological stimuli, such as speech, neural oscillations can synchronize in time ranges from the level of phonemes to the level of the syllables (for a review, see Ahissar et al., 2001; Giraud and Poeppel, 2012; Saoud et al., 2012; Power et al., 2013), with differential synchronization abilities of hemispheres potentially underlying the hemispheric specialization for speech (Giraud et al., 2007). Although such neural entrainment occurs across various oscillatory frequencies (Gross et al., 2013; Peelle et al., 2013), it is most frequently observed for low frequencies (Luo and Poeppel, 2007; for review see, Peelle and Davis, 2012). Moreover, synchronization seems to depend on previous exposure to a speech cue. The degree of familiarity with speech can facilitate entrainment (Lidji et al., 2011) and modulate oscillatory responses. Power synchronization in the theta band was observed when listening to the native language only (Pérez et al., 2015) and increased gamma-band power was observed when listening to the native language compared to a foreign language (Peña and Melloni, 2011). This indicates that neural oscillations might help to assess the meaning of speech.

Another potential neural correlate of sensory entrainment is *neural activation in the dorsal striatum*. Several studies manipulating the temporal context of auditory sequences have reported activation in the putamen. Typically, this activation was observed when experimental subjects listened to sound sequences comprising temporal regularity. These studies examined explicit processing of timing by applying perceptual tasks, such as regularity detection (Grahn and Rowe, 2009) and duration discrimination in the context of a temporally regular sequence (Teki et al., 2011a), motor tasks such as the reproduction of a rhythm comprising temporal regularity or motor synchronization with the beat (Riecker et al., 2003; Chen et al., 2008), or simply listening to a rhythmic beat (Grahn and Brett, 2007). Hence, models of auditory perception have attributed a central role to the basal ganglia, for example, as a brain region tracking temporal modulations in acoustic signals including speech (Kotz et al., 2009; Teki et al., 2011b; Schwartze et al., 2012) or integrating predictive coding in speech perception (Lim et al., 2014).

Although the above evidence indicates that activation in the putamen plays a role in temporal regularity perception, it does not reveal whether the putamen plays a role in sensory entrainment. We measured activation in the putamen in a typical sensory entrainment task (Geiser et al., 2012). Participants had to detect an intensity change in a sequence of tones that were either temporally regular (isochronous) or temporally irregular. As expected, temporal regularity enhanced auditory perception for tone intensity, and there were two associated patterns of brain activation. First, there was decreased activation in bilateral regions of the temporal lobe in response to temporally regular sequences compared to irregular sequences. Second, there was increased activation in the putamen in response to temporally regular sequences relative to irregular sequences. Thus, striatal activation is not only involved when participants encounter temporal regularity but is observed in a typical sensory entrainment task. Importantly, across individuals, the reduced activation in primary, and secondary auditory cortices in response to temporal regularity perception, which yielded better behavioral performance, was linearly correlated with increased activation in the putamen. This correlation could indicate that the striatum dynamically interacts with the sensory cortex either directly or through a mediating brain area to facilitate perception in the context of sensory entrainment.

The functional role that the striatum could play in sensory entrainment remains elusive. One could imagine that the putamen simply detects temporal regularity or the average tempo of a sequence. Alternatively, the putamen may crucially underlie sensory entrainment by internally perpetuating temporal regularity and predicting future acoustic events. Evidence demonstrating the latter is still lacking. However, when participants explicitly tracked temporal regularity in the second of two sequences in which the tempo either changed or did not change between the two sequences, greater activation in the putamen was found when a sequence repeated the tempo of a previously heard sequence than when the tempo changed (Grahn and Rowe, 2013). This indicates that the striatum responds when a tempo prediction is confirmed by the external stimulus. Authors suggest that this indicates the encoding of predictive aspects of temporal regularity perception. This is in line with an earlier study suggesting that the putamen encodes prediction, at least in motor learning (Haruno and Kawato, 2006). Further studies will need to test whether putamen activation in the context of sensory entrainment is related more to the confirmation of a prediction or to the generation of a prediction.

Whether the two neural correlates of sensory entrainment, neural oscillations and striatal activation, are functionally linked remains to be investigated. However, evidence from motor studies suggests a potential link. At least in some putaminal recording sites, the spectral power of beta oscillations increases when monkeys perform self-generated tapping in a previously learned tempo compared to when they tapped in response to an irregularly appearing cue production (Bartolo et al., 2014; Bartolo and Merchant, 2015). This indicates that some striatal circuits might play a role in the internal generation of temporal regularity, at least in the context of motor processing. Thus, it is possible that increased putamen activation as measured in the BOLD response is driven by enhanced putaminal beta activity.

## Is attention necessary for sensory entrainment?

It has long been known that “dynamic attending” induced by temporally regular stimuli can lead to faster reaction times to temporally expected points in time (Jones and Boltz, 1989; Barnes and Jones, 2000; London, 2004). Most recent experimental paradigms measuring sensory entrainment comprise active tasks in which participants focus their attention on the entraining stimulus, allowing stimulus-driven attending that involves temporal expectancy (Jones et al., 2002; Sanabria and Correa, 2013). Sensory attenuation and putaminal activation in the context of sensory entrainment is observed in the presence of endogenous attention (Lange, 2010; Costa-Faidella et al., 2011; Geiser et al., 2012), and synchronization of neural oscillations to sensory stimuli is particularly strong when attention is directed toward the entraining sound (Besle et al., 2011; Horton et al., 2013).

While the sensory effect of temporal context in the presence of endogenous attention is well investigated, less is known about temporal expectancy in the absence of endogenous attention. Evidence from visual studies suggests that temporal expectation and attention might influence neural activation in opposite ways (Summerfield and Egner, 2009; Kok et al., 2012; see also Arnal and Giraud, 2012). In the auditory domain, orthogonal manipulation of expectation and attention showed an attenuation effect on the N1 in the attended condition only (Hsu et al., 2014). Based on this finding, one could hypothesize that the attenuating effect of a regular temporal context might depend on the presence of endogenous attention.

However, neural effects of entrainment are also observed in the absence of endogenous attention. In passive oddball paradigms, temporal predictability influences auditory ERPs to acoustic (Geiser et al., 2010) or higher-level deviants (Tavano et al., 2014). Moreover, neural oscillations entrain to auditory stimuli when participants' endogenous attention is directed to a concurrent visual (Fujioka et al., 2009, 2012) or auditory stimulus (Golumbic et al., 2013; Horton et al., 2013; Rimmele et al., 2015). Moreover, in an unattended condition, expectation modulates auditory beta-band synchronization to tones (Todorovic et al., 2015). Thus, attention networks use oscillatory phase entrainment for both enhancement and suppression of auditory signals (for a review, see Calderone et al., 2014).

The above evidence indicates that sensory entrainment is influenced by attention but that neural effects of entrainment are present in both attended and unattended processing conditions. Further studies will need to investigate the behavioral effects and the cortico-striatal mechanisms related to sensory entrainment as a function of attention.

*In summary*, sensory entrainment is essential for auditory perception. It drives perception to be best at temporally expected moments in time. Neural oscillations and, potentially, striatal brain activation underlie sensory entrainment. Whether these two correlates are part of the same mechanism and the way in which attention interacts with mechanisms of sensory entrainment remain to be investigated.

## Author contributions

Conceptualization, EG. Writing-Original Draft, EG, CS. Writing, Review, and Editing, EG, CS. Visualization, CS.

## Funding

Swiss National Science Foundation: PZ00P1_148184/1 awarded to EG and FN320030-159708 awarded to Stephanie Clarke.

### Conflict of interest statement

The authors declare that the research was conducted in the absence of any commercial or financial relationships that could be construed as a potential conflict of interest.
